# Magnetic Resonance Imaging Image Segmentation Under Artificial Intelligence Neural Network for Evaluation of the Effect of Butyphthalide Combined With Edaravone on Neurological Function in Patients With Acute Cerebral Infarction

**DOI:** 10.3389/fnbot.2021.719145

**Published:** 2021-09-29

**Authors:** Bin Li, Guoping Liu

**Affiliations:** ^1^Department of Neurology, The Third Affiliated Hospital of South China University, Hengyang, China; ^2^Department of Gastroenterology, The Third Affiliated Hospital of South China University, Hengyang, China

**Keywords:** artificial intelligence neural network, convolutional neural network, magnetic resonance, image segmentation, butyphthalide, edaravone, acute cerebral infarction, neural function

## Abstract

This research was developed to investigate the effect of artificial intelligence neural network-based magnetic resonance imaging (MRI) image segmentation on the neurological function of patients with acute cerebral infarction treated with butylphthalide combined with edaravone. Eighty patients with acute cerebral infarction were selected as the research subjects, and the MRI images of patients with acute cerebral infarction were segmented by convolutional neural networks (CNN) upgraded algorithm model. MRI images of patients before and after treatment of butylphthalide combined with edaravone were compared to comprehensively evaluate the efficacy of this treatment. The results showed that compared with the traditional CNN algorithm, the running time of the CNN upgraded algorithm adopted in this study was significantly shorter, and the Loss value was lower than that of the traditional CNN model. Upgraded CNN model can realize accurate segmentation of cerebral infarction lesions in MRI images of patients. In addition, the degree of cerebral infarction and the degree of arterial stenosis were significantly improved after treatment with butylphthalide and edaravone. Compared with that before treatment, the number of patients with severe cerebral infarction or even vascular stenosis decreased significantly (*P* < 0.05), and gradually changed to mild vascular stenosis, and the neurological dysfunction of patients was also significantly improved. In short, MRI image segmentation based on artificial intelligence neural network can well-evaluate the efficacy and neurological impairment of butylphthalide combined with edaravone in the treatment of acute cerebral infarction, and it was worthy of promotion in clinical evaluation of the treatment effect of acute cerebral infarction.

## Introduction

Acute cerebral infarction refers to the sudden interruption of blood supply in the brain, which then leads to the necrosis of brain tissue (Lyu et al., 2019). The onset of the disease is sudden, usually during quiet rest or sleep, and the onset peaks in a few hours or within 1 to 2 days. Usually, headache, dizziness, tinnitus, hemiplegia, difficulty swallowing, slurred speech, nausea, vomiting, etc. can occur. The patient will be unconscious in severe cases (Zhang et al., 2019). In clinical drug therapy, butylphthalide and edaravone are the most common drugs. Butylphthalide can improve the injured central nervous function in patients with acute ischemic stroke (Zhou and Kou, 2019; Pan et al., 2020). Animal experiments revealed that butyphthalide played an anti-cerebral ischemic effect by blocking multiple pathological links of brain damage caused by ischemic stroke (Chen et al., 2019). Butyphthalide significantly improves the microcirculation and blood flow in the ischemic brain area. It can increase the number of capillaries in the ischemic area, reduce the infarct area of local cerebral ischemia, reduce cerebral edema, and inhibit nerve cell apoptosis. In addition, it also has anti-cerebral thrombosis and anti-platelet aggregation effects. Edaravone is a kind of brain protective agent, which belongs to free radical scavenger. It can prevent the progression of cerebral edema and cerebral infarction, relieve the accompanying neurological symptoms, and inhibit delayed neuronal death (Tan et al., 2019). Mechanism studies suggested that edaravone can scavenge free radicals and inhibit lipid peroxidation, so as to inhibit the oxidative damage of brain cells, vascular endothelial cells, and nerve cells. Moreover, clinical practice suggested that the effect of the combined treatment of the two medicines was much higher than that of the single treatment, which had a positive effect on effectively curbing the progression of the disease and delaying the progress of the disease (Li et al., 2020).

Clinically, the treatment effect of the disease is often evaluated by head CT, cerebral angiography, etc. However, CT scans can't clearly visualize the brainstem and cerebellum lesions, and cerebrovascular angiography also has certain injuries and complications, so their clinical use is limited (Wo et al., 2020). Magnetic resonance imaging (MRI) is a type of tomography, which harvests electromagnetic signals from the human body and reconstruct human body information under magnetic resonance phenomena. It has been widely used in imaging diagnosis of various systems throughout the body. It has good soft tissue resolution and multi-directional imaging function. Based on the adoption of dynamic enhancement and fat suppression technology, it can accurately display the extent of the lesion, the edge, the involvement of surrounding tissues, the relationship with surrounding tissues, and the blood perfusion of the lesion, so as to accurately stage the disease. Moreover, it can also show large occluded blood vessels, which makes up for the deficiencies of other examinations, and has guiding significance for the clinical determination of treatment plans (Drazyk et al., 2019). The precise segmentation of the infarct lesion during the examination is particularly important for the objective examination of the treatment effect, and it is also very important for the follow-up evaluation (Huang et al., 2014). MRI image diagnosis can cause a certain degree of misdiagnosis due to noise and inaccurate image segmentation, while convolutional neural network (CNN) has achieved remarkable results in target recognition and biological image segmentation in recent years (Chen et al., 2020). Therefore, in this study, MRI images were segmented based on CNN algorithm to comprehensively evaluate the efficacy of butylphthalide combined with edaravone in patients with acute cerebral infarction and its influence on neurological function. The results of the study were reported as follows.

## Materials and Methods

### Basic Information

Eighty patients with acute cerebral infarction admitted to hospital from January 2018 to June 2020 were recruited as the research objects, including 56 males and 24 females. The age range was 44–71 years old, with an average age of (50.54 ± 2.34) years old. In terms of complications, 51 people were with hypertension, 27 people were with diabetes, 18 people were with hyperlipidemia, and 47 people were with coronary heart disease. The shortest course of the disease was one month, and the longest time was 10 years. Magnetic resonance examination was performed for all patients. Diagnostic criteria: the relevant diagnostic criteria for acute cerebral infarction in the Diagnostic Criteria for Cerebral Infarction was referred to (Song et al., 2012). Inclusion criteria: those who met the above diagnostic criteria; those who with complete clinical data and records; those who were not allergic to the relevant treatment and test drugs used in the treatment or examination in this study; patients and their family members were aware of the study process and signed the informed consent forms. Exclusion criteria: those with cognitive impairment; those with poor compliance and unable to cooperate with the researcher; those who had recently received relevant medications; and those with congenital heart disease. This experiment had been approved by the ethics committee of hospital, and the patients had signed the informed consent forms.

### MRI Examination

The patient was examined with a magnetic resonance diagnostic instrument (manufacturer: Siemens Avanto, Germany; model: Optima 360), and the body was imaged with a phased array surface coil.

The examinee took a supine position and put both hands on both sides of the body. The body coil was wrapped around the desired scanning range so that it completely covered the target area. A single voxel point was adopted to resolve the spin echo sequence. The parameter settings were as follows. The echo time was 1,500 ms, repetition time was 470 ms, and the voxel size was 21 × 20 × 21 mm. The echo time of FSE/T2WI sequence scan was 130 ms, and the repetition time was 4,350 ms. The echo time of the reverse recovery sequence scan was 20 ms, and the repetition time was 5,635 ms. The echo time of the axis FSE/T2WI sequence scan was 100 ms, and the repetition time was 4,475 ms. The scan layer spacing and layer thickness were both set to 5.2 mm. It took about 6–8min to finish the regular scan. The patient's state was observed, and the scan would be stopped immediately and corresponding treatment would be given if there was any abnormality.

### MRI Image Segmentation Based on CNN

**Figure 1:**
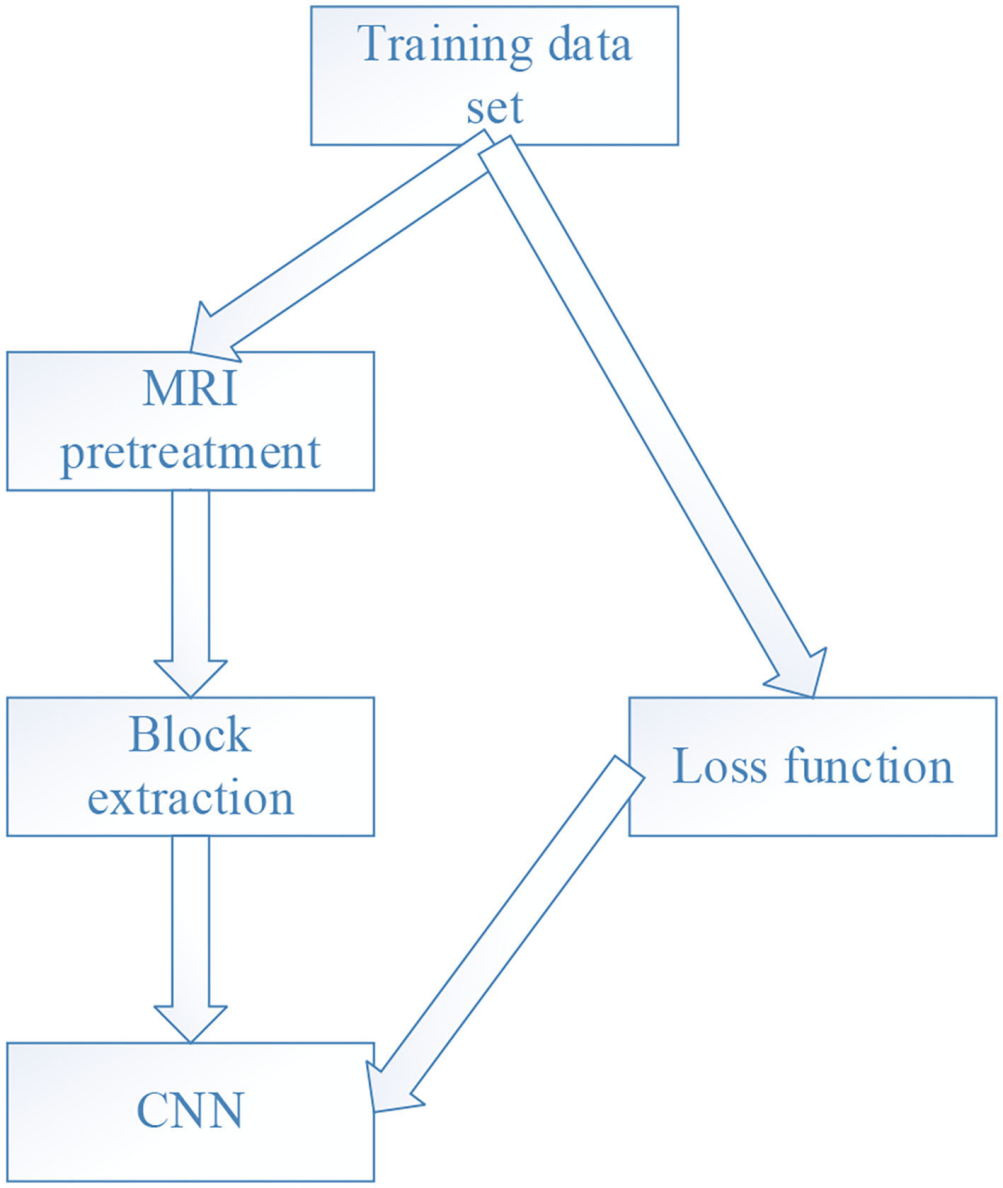
CNN algorithm flow.

CNN is a machine learning model (Figure 1), which has strong adaptability and has a good extraction effect on local features of data. Its main components include data input layer, convolution layer, activation function, other processing layers, and output layer.

The input layer mainly accepts input data. Usually, the input layer is multi-dimensional, which can completely retain the structural information of the data itself in the picture. The convolutional layer uses a convolution kernel to extract features from the first input data. Usually, the convolution kernel changes with the depth of the network. Basic information such as lines and contours are extracted after convolution, and this information is determined by the position of the convolutional layer in the network. The deeper the convolutional layer, the larger the receptive field. Therefore, the more local features considered by fusion, the more abstract the extracted features. First, the statistics of the characteristics of the noise are carried out, and the approximate model of the noise is as follows.


(1) $$\begin{array}{l} pi=pi-1⊚qi+ai \end{array}$$

In equation (1), pi-1 represents the input of the first layer, ⊚ represents the convolution operation, qi is the number of convolutional layers, and ai represents the bias. The extracted feature map pi is the output of the first layer.

The activation function is an important part of the deep CNN, which introduces non-linear operations into the entire model. If there is no activation function, superimposing other linear operation layers to increase the depth of the network will make the expressiveness of the entire network worse. Different activation functions will get different gradient derivatives when the network is backpropagated. The specific operations in the development of deep learning networks are as follows.


(2) $$\begin{array}{l} y=1/\left(1+e-i\right) \end{array}$$

(3) $$\begin{array}{l} \text{tanh}\left(i\right)=\left(1-e-2i\right)/\left(1+e-2i\right) \end{array}$$

(4) $$y=\{\begin{array}{cc} i,\; & i\geq 0\\ 0,\; & i0 \end{array}$$

Pooling refers to the function that performs a local pooling operation on the feature map, which can remove part of the redundant information, reduce the feature map, reduce the amount of calculation, and maintain the invariance of the position of the feature in the image. It can also help the model prevent overfitting. The existing general pooling operation window areas do not overlap, such as the commonly used average pooling and maximum pooling. Overlapping pooling, as the name implies, is that the areas of adjacent pooling operations overlap. There is also pyramid pooling, which can convert image convolutional feature pooling of any scale into the same dimension. Each node in the fully connected layer is connected to all the nodes in the previous layer, and is used to synthesize the features extracted from the front and output them to the output layer. Due to its fully connected characteristics, generally, the parameters of the fully connected layer are also the most. The entire training process refers to calculating this round of loss according to the loss function after input of the previous propagation through all levels and processing, and then propagating the loss backwards. Appropriate optimization methods such as stochastic gradient descent are utilized to update the parameters of each layer in the direction of reducing the loss.

The image is preprocessed firstly. Since the MRI image will change with the distortion of the Bayesian field, the gray value of the image appears uneven. To change this phenomenon, it is necessary to learn a set of gray-scale markers from each sequence in the training set. The definition of this set is as follows.


(5) $$\begin{array}{l} P_{I}=\left\{sv_{1},p_{s10},p_{s20},...p_{s90},sv_{2}\right\} \end{array}$$

*sv*_1_ and *sv*_2_ are selected from each MRI sequence, and *p*_*s*_ represents the gray value in percentile *I*. The histogram of each sequence will become closer after training.

**Figure 2:**
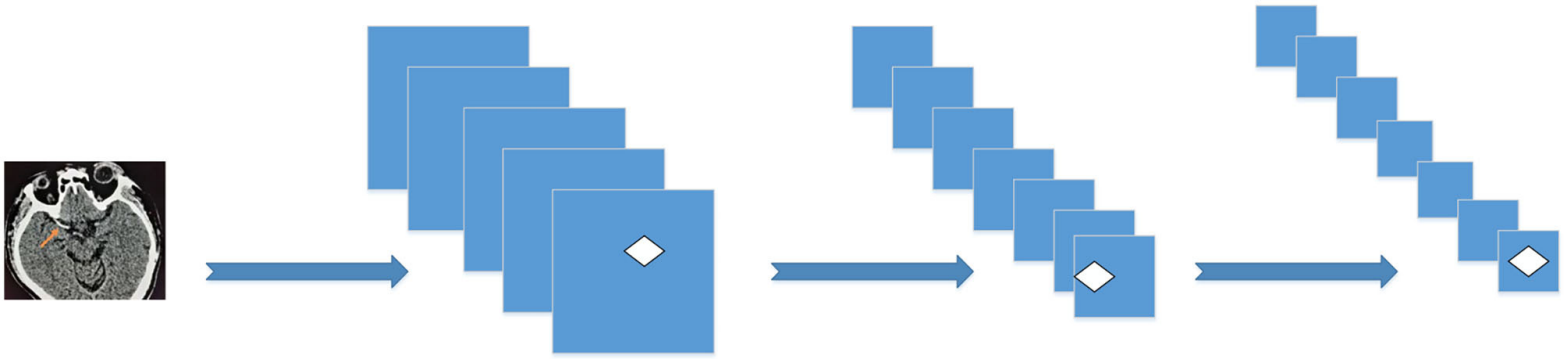
CNN model.

The CNN model constructed in this research is shown in Figure 2.

The low-level features are transformed into high-level features through multi-layer learning. The obtained high-level features are input into the Softmax classifier through intermediate preprocessing. Each pixel in the image is classified into two categories, and the probability of whether it belongs to the cerebral infarction focus or the normal brain tissue is obtained. The classification is based on the probability of the category, so as to obtain the segmented binary image of the infarct focus.

### Evaluation Criteria for the Degree of Arterial Stenosis and Neurological Deficit

In this study, the diagnostic criteria used to determine the severity of acute cerebral infarction patients were as follows. When the diameter of the arteries in the MRI images decreased by 0 to 49% compared with the normal ones, or when the vascular signal did not exist, the patients were judged to be mild stenosis. Moderate stenosis was defined when the arterial diameter of the patient on MRI was 50 to 69% less than that of the normal person, or when the vascular signal loss was 50 to 69%. Patients with severe stenosis were judged to have a 70–99% reduction in arterial vessel diameter or 70–99% loss of vessel signal in MRI images compared with normal subjects. If the vascular lumen can't be completely shown and the vascular signal was completely lost during the MRI scan, the arterial obstruction was determined.

In addition, the neurological deficiency scale (NDS) was used to assess neurological deficits in patients with acute cerebral infarction via the national institutes of health stroke scale (NIHSS).

### Statistical Methods

SPSS 19.0 was employed for data statistics and analysis. Mean ± standard deviation (x ± s) was how measurement data were expressed. *T* test was used to compare the mean values among all groups, and statistical data were expressed as percentage (%) and χ^2^ test was adopted. *P* < 0.05 was used to indicate statistically significant differences.

## Results

### Test Results of CNN Upgraded Model

**Figure 3:**
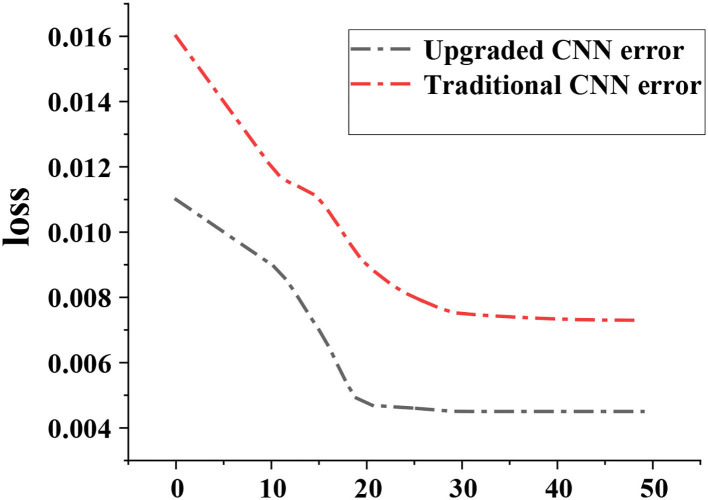
Comparison of the Loss curves of the two CNN algorithms.

Figure 3 showed the comparison of Loss curves of two CNN algorithms. The results show that the Loss curves of both the traditional CNN model and the upgraded CNN model tended to converge gradually with the increase of the number of iterations. Loss values of the upgraded CNN model were all smaller than those of the traditional CNN model.

### Reconstruction Time Analysis of CNN Model

**Figure 4:**
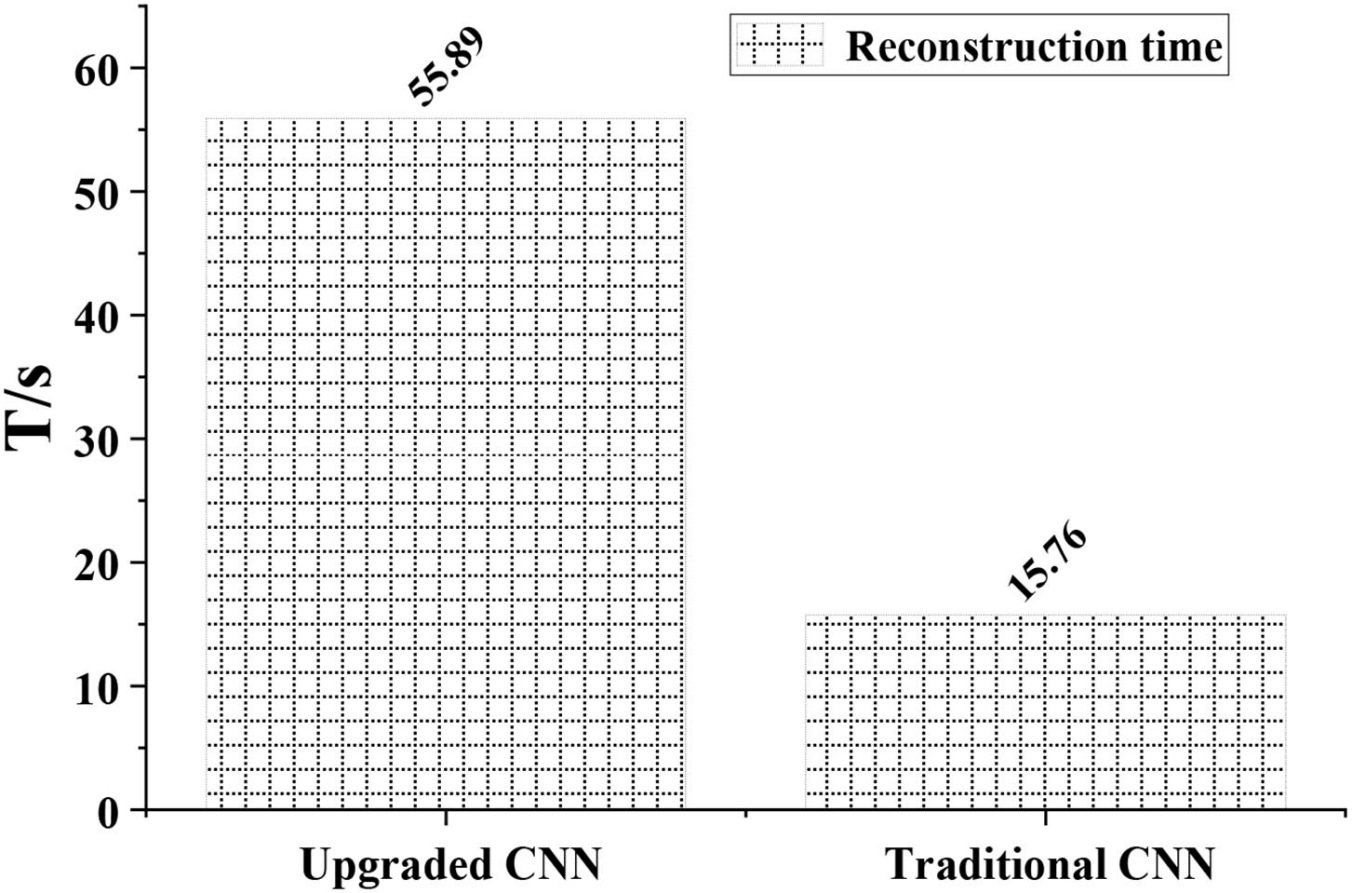
Analysis of reconstruction time of CNN model.

Figure 4 showed the reconstruction time of the CNN model. The reconstruction time of the traditional CNN algorithm was 55.89 s, and that of the DRCNN algorithm was 15.76 s. The difference between the two was significant (*P* < 0.05).

### Comparison of Patient MRI Images Before and After CNN Algorithm Processing

**Figure 5:**
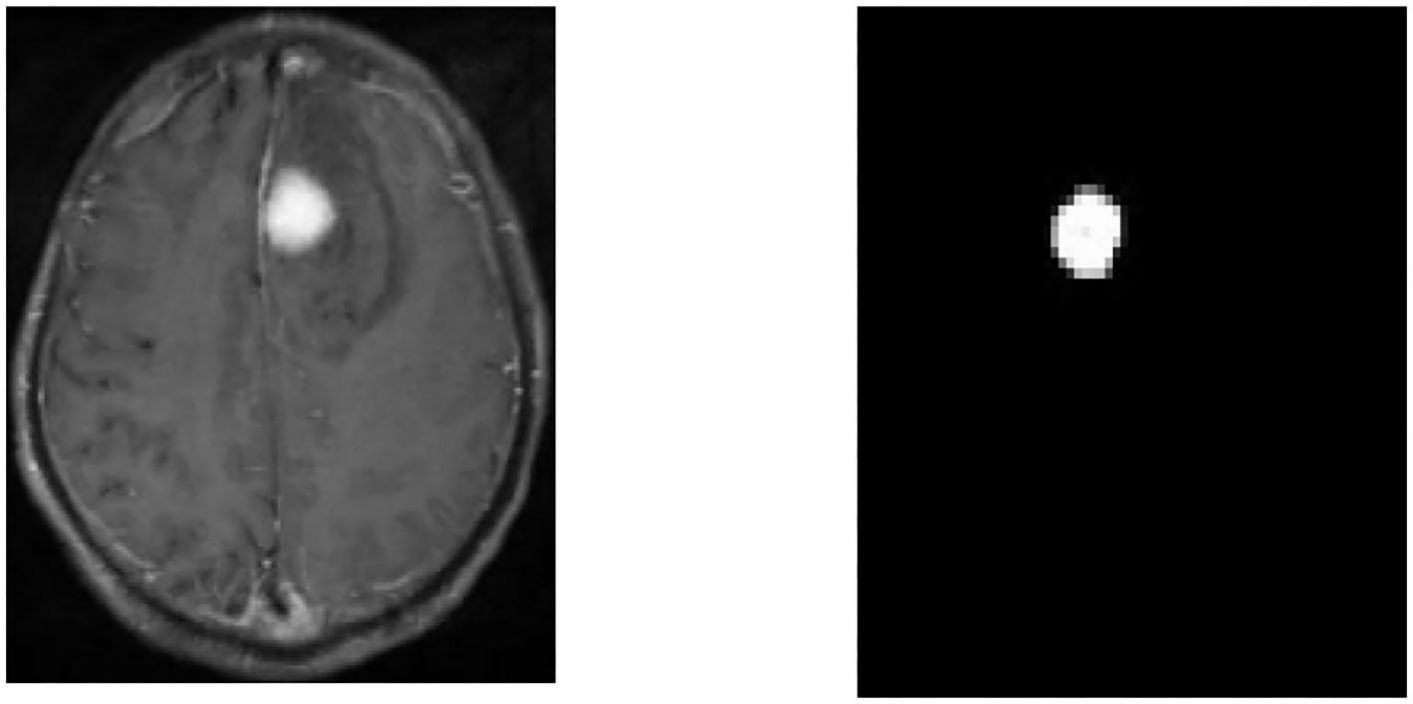
Segmentation results. MRI image of the patient **(left)** and segmentation result image **(right)**.

Figure 5 showed that the MRI image segmentation under the CNN algorithm can completely segment the cerebral infarction lesions, and the effect was good.

### Comparison of MRI Images of Patients Before and After Treatment

**Figure 6:**
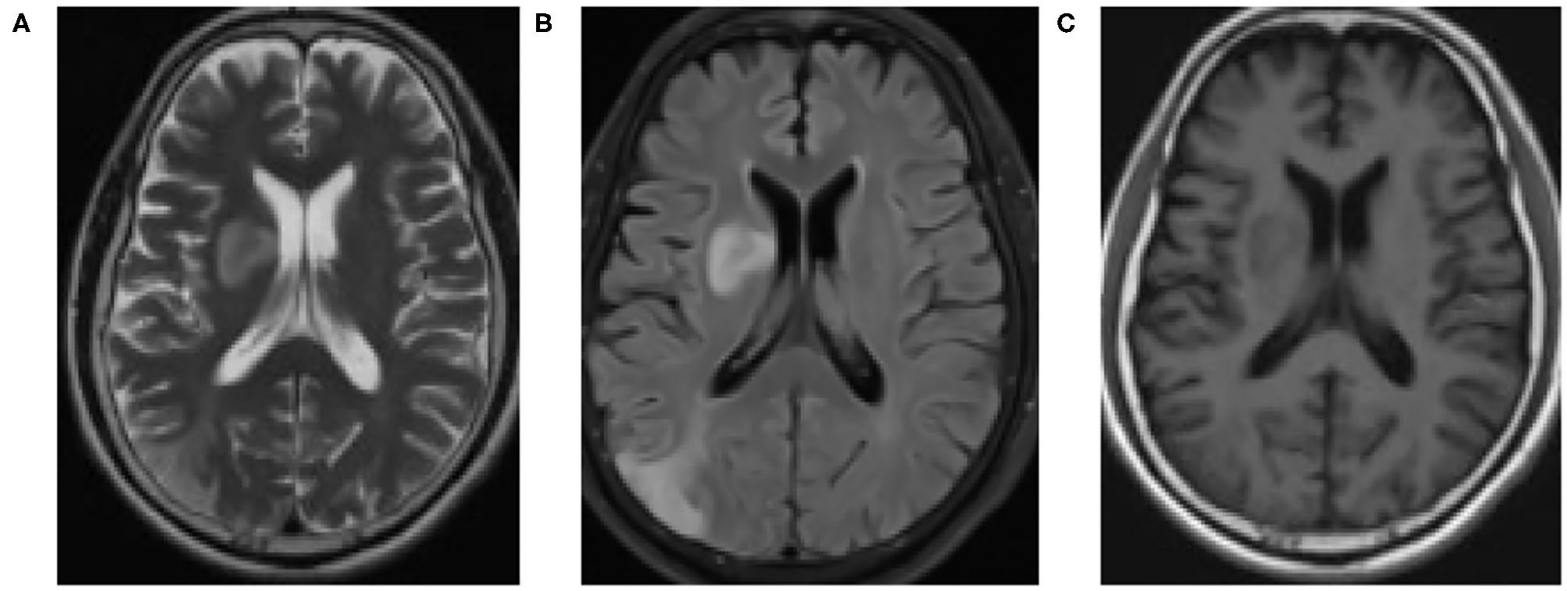
MRI examination results. **(A)** before drug treatment; **(B)** during drug treatment; **(C)** one week after drug discontinuation.

Figure 6 showed the comparison of MRI images of patients before and after treatment. MRI results showed that with the progression of butylphthalide combined with edaravone treatment, the cerebral infarction area of patients with acute cerebral obstruction gradually decreased. Signals also gradually recovered in areas where the arteries were blocked.

### Degree of Arterial Stenosis

**Figure 7:**
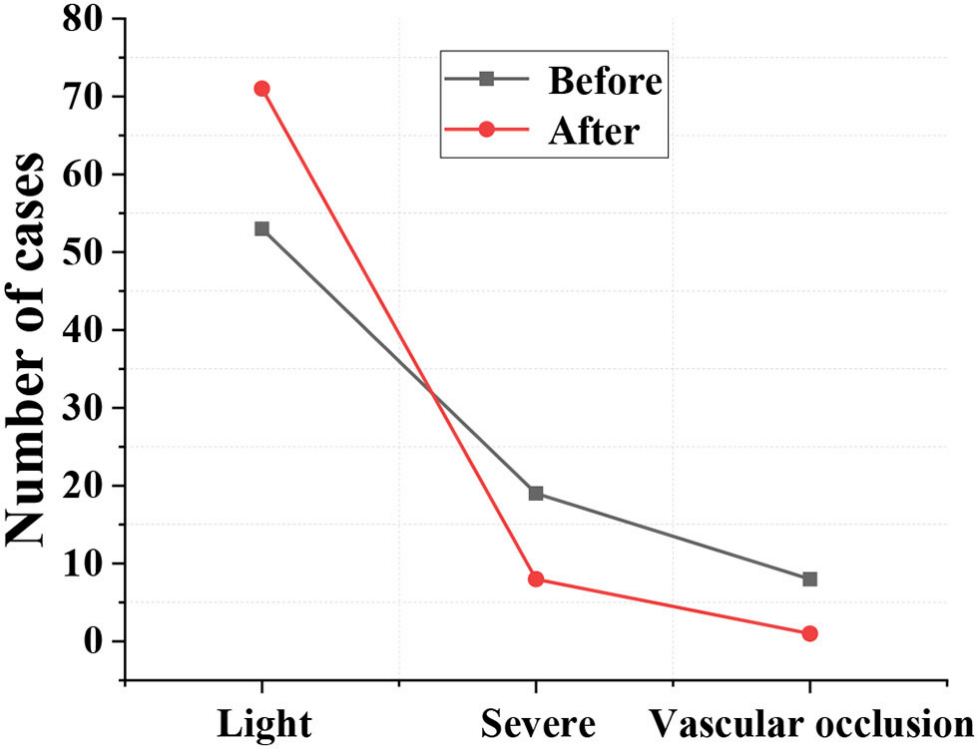
Degree of arterial stenosis.

Figure 7 showed that the number of patients with severe cerebral infarction and even vascular occlusion after treatment decreased compared with that before treatment, gradually turning to mild, with substantial difference (*p* < 0.05).

### Degree of Neurological Deficit

**Figure 8:**
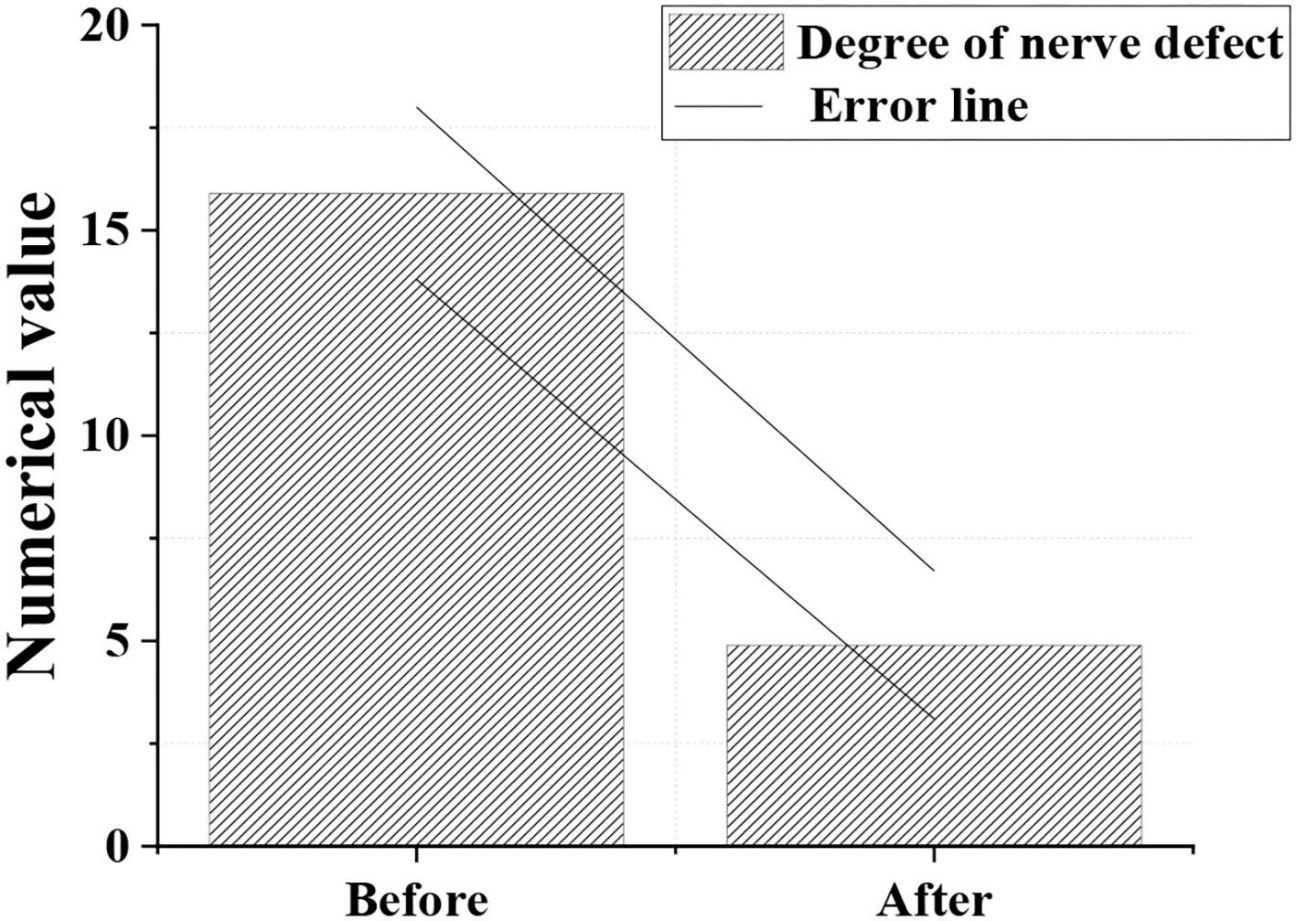
Comparison of degree of neurological deficits.

Figure 8 illustrated that the degree of neurological deficit after treatment was significantly reduced compared with before treatment, with remarkable difference (*p* < 0.05).

## Discussion

Acute cerebral infarction is a common neurological disease, whose occurrence and development involve a series of ischemic chain reactions (Taomoto et al., 2010; Liu et al., 2015; Zhao et al., 2018). Current studies confirmed that both butylphthalide and edaravone treatment can effectively protect the cerebral nerves by increasing the level of related ions in cerebrovascular endothelium and reducing the release of oxygen free radicals (Zhao et al., 2005; Du et al., 2015). In addition, edaravone treatment can also increase the number of capillaries in the ischemic area, establish new collateral circulation, and improve brain energy metabolism, which has obvious clinical therapeutic effects (Zhen et al., 2011). At present, MRI is the main definite examination method for the diagnosis and differential diagnosis of central nervous system diseases (Pei et al., 2001). Clinically, MRI is often selected for the diagnosis of acute cerebral infarction, but this method may be misdiagnosed to some extent due to noise and other reasons (Abe et al., 2013). Therefore, in this study, the MRI image segmentation based on CNN algorithm was used to process the MRI images of patients with acute cerebral infarction, so as to comprehensively evaluate the therapeutic effect of butylphthalide combined with edaravone in the treatment of acute cerebral infarction.

The results showed that the CNN algorithm and DRCNN algorithm adopted in this study significantly shortened the running time of the model compared with the traditional CNN algorithm. Accurate segmentation of cerebral infarction lesions in MRI images of patients with acute cerebral infarction was realized. Moreover, after treatment with butylphthalide combined with edaravone, the degree of cerebral infarction and the degree of arterial stenosis were significantly improved. Compared with that before treatment, the number of patients with severe cerebral infarction or even vascular stenosis was significantly reduced (*P* < 0.05), with a gradual shift to mild vascular stenosis and a marked improvement in neurological dysfunction. It was consistent with the findings of Chen et al. (2021). It was verified that the MRI image segmentation technology based on CNN algorithm can perform accurate segmentation of acute cerebral infarction lesions and improve the MRI image diagnosis effect of acute cerebral infarction. It can clearly present the patients' lesions before and after treatment. It was also suggested that butylphthalide combined with edaravone had a good therapeutic effect on acute cerebral obstruction and can significantly improve the condition of cerebral obstruction and arterial stenosis in patients. To a certain extent, the nerve function of the patients was restored.

## Conclusion

In this study, CNN algorithm model was established and applied to the MRI image segmentation of patients with acute cerebral infarction to evaluate the effect of butylphthalide combined with edaravone treatment on the neurological function of patients. The results showed that MRI images based on CNN algorithm could clearly show the lesions of patients with acute cerebral infarction. In addition, it was observed that after the treatment of butylphthalide combined with edaravone, the lesion of cerebral infarction became smaller and the condition of artery stenosis gradually improved. These results indicated that CNN-based MRI image segmentation had a high application value in the evaluation of acute cerebral infarction after drug treatment. However, the shortcomings of this study are that the included sample size is small, the source is single, and the classification and comparison of patients with acute cerebral infarction at different stages are not completed. Therefore, further studies will be carried out in the future work, and the application of CNN-based MRI image segmentation to the therapeutic effect evaluation of other brain diseases will be considered. In conclusion, MRI image segmentation based on artificial intelligence neural network can effectively evaluate the treatment effect of acute cerebral infarction, which provides a certain reference for the clinical evaluation of the treatment effect of acute cerebral infarction.

## Data Availability Statement

The original contributions presented in the study are included in the article/supplementary material, further inquiries can be directed to the corresponding author/s.

## Author Contributions

BL: writing-original draft, conceptualization, supervision, and formal analysis. GL: writing-review & editing, methodology, and software. All authors contributed to the article and approved the submitted version.

## Conflict of Interest

The authors declare that the research was conducted in the absence of any commercial or financial relationships that could be construed as a potential conflict of interest.

## Publisher's Note

All claims expressed in this article are solely those of the authors and do not necessarily represent those of their affiliated organizations, or those of the publisher, the editors and the reviewers. Any product that may be evaluated in this article, or claim that may be made by its manufacturer, is not guaranteed or endorsed by the publisher.
